# Exploring the Adaptations of the Free Maternity Policy Implementation by Health Workers and County Officials in Kenya

**DOI:** 10.9745/GHSP-D-23-00083

**Published:** 2023-10-30

**Authors:** Boniface Oyugi, Sally Kendall, Stephen Peckham, Stacey Orangi, Edwine Barasa

**Affiliations:** a M and E Advisory Group, Nairobi, Kenya.; b Centre for Health Services Studies, University of Kent, Canterbury, United Kingdom.; c Health Economics Research Unit, KEMRI-Wellcome Trust Research Programme, Nairobi, Kenya.; d Center for Tropical Medicine and Global Health, Nuffield Department of Medicine, University of Oxford, Oxford, United Kingdom.

## Abstract

**Background:**

In 2017, Kenya launched the free maternity policy (FMP) that aimed to provide all pregnant women access to maternal services in private, faith-based, and levels 3–6 public institutions. We explored the adaptive strategies health care workers (HCWs) and county officials used to bridge the implementation challenges and achieve the FMP objectives.

**Methods:**

We conducted an exploratory qualitative study using Lipsky's theoretical framework in 3 facilities (levels 3, 4, and 5) in Kiambu County, Kenya. The study involved in-depth interviews (n=21) with county officials, facility in-charges and HCWs, and key informants from national and development partner agencies. Data were audio-recorded, transcribed, and analyzed using a framework thematic approach.

**Results:**

The results show that HCWs and county officials applied several strategies that were critical in shaping the policymaking, working practice, and professionalism and ethical aspects of the FMP. Strategies of policymaking: hospitals employed additional staff, and the county developed bylaws to strengthen the flow of funds. Strategies of working practice: hospitals and HCWs enhanced patient referrals, and facilities enhanced communication. Strategies of professionalism and ethics: nurses registered and provided service to mothers, and facilities included employees in planning and budgeting. Maladaptations included facilities having leeway to provide FMP services to populations who were excluded from the policy but had to bear the costs. Some discharged mothers immediately after birth, even before offering the fully costed policy benefits, to avoid incurring additional costs.

**Conclusions:**

The role of policy implementers and the built-in flexibility and agility in implementing the FMP could enhance service delivery, manage the administrative pressures of implementation, and provide mothers with personalized, responsive service. However, despite their benefits, some resulting unintended consequences may need interventions.

## Abstract (precis)

To achieve the objectives of the free maternity policy in Kenya and overcome implementation challenges, health care workers and county officials—as policy implementers—covertly and unofficially developed local arrangements and adaptive strategies.

## Teaser

**Key Findings:**

- The health care workers and county officials developed 3 adaptive strategies for implementing the free maternity policy that were critical in meeting the policy objectives:
  - Mitigating bureaucratic processes to employ more workers and innovating complementary programs to support the utility of services
  - Enhancing working practices to improve referrals, clients' policy knowledge, and client communication on the policy requirements
  - Enhancing health care workers' professionalism and ethics by registering more mothers to uptake services, providing services to vulnerable populations excluded from the official written policy, and improving the claims process
- The strategies created unintended negative consequences, such as health care workers' workload increasing and facilities discharging mothers immediately after birth, putting them at risk of complications.

**Key Implication:**

- Enhancing the roles of health care workers and county officials in policy implementation can build the flexibility and agility of the implementation process, leading to more services being provided to more mothers. Further analysis of their professional resilience in public services planning needs additional theoretical and empirical attention.

## INTRODUCTION

Reducing and eliminating pregnancy-related mortality remains a priority to achieving the Sustainable Development Goals. Despite Kenya's maternal mortality ratio decreasing from 564 to 530 from 2000 to 2020^1^ and the neonatal mortality rate reducing from 33 to 21 deaths per 1,000 live births between 2003 to 2022,^2^ mothers and neonates still die from preventable pregnancy-related complications.^3^ Kenya has had various health sector reforms that have aimed to enhance the utilization and quality of maternity care and reduce maternal and neonatal mortalities.^4^^–^^6^

In 2013, the Kenyan government initiated free maternity and primary health care services,^4^ which increased the utilization of maternity services^7^ but faced implementation challenges of poor service delivery because of lack of adequate preparation before implementation.^8^ To overcome the implementation challenges, the country transitioned to a new expanded free maternity policy (FMP) in 2017 that aimed to provide access to maternal services to all pregnant women in private, faith-based, and all level 3–6 public institutions.^9^^,^^10^ The new FMP, called *Linda Mama* (Swahili for “caring for the mother”), was managed through the National Hospital Insurance Fund (NHIF).^9^ The National Treasury provided the funds to the Ministry of Health (MOH), which then provided the funds to the NHIF. The NHIF distributed the funds through the county treasury directly to private and faith-based health facilities or indirectly to public facilities as payment for services provided (e.g., such as antenatal care, delivery, and postnatal care).^10^ After the mothers used the services, the facilities submitted claims for reimbursement to the NHIF.

Kenya has a devolved system of governance in which the national government is responsible for regulatory and policy guidance, capacity-building, and providing oversight to the referral system, and 47 semiautonomous county governments are responsible for service delivery.^11^^,^^12^ Health care service provision and financing is done by both the public and private sectors. The public health care sector operates through a tiered system. Level 1 forms the community units overseen by community health workers whose role of providing promotive services (health education, treating minor ailments, and identifying cases that require referral to health facilities) are guided by the Kenya Community Health Policy 2020–2030.^13^ Both level 2 (dispensaries) and level 3 (health centers) provide primary health care services in addition to coordinating the community in their areas of jurisdiction. Levels 4 and 5 offer curative services as county secondary referral facilities in addition to some being training centers. Level 6 facilities are semiautonomous tertiary facilities offering specialized care and serving as training institutions.

As part of evaluations of the Kenyan FMP, to date, scholars have focused on understanding its immediate and trend effect,^5^ its impacts on mortality and utilization of services,^14^^–^^16^ and the resulting quality of care aspects—both provision and experience.^17^^,^^18^ However, by only focusing on these aspects, our understanding of the implementation of the FMP, especially as conducted by policy implementers, is limited. One study evaluated the FMP implementation process and highlighted its challenges around the implementation fidelity issues, such as misunderstanding inclusion criteria at the implementation, poor access to some services/benefits packages, mothers incurring out-of-pocket payments, claims process management challenges, financial arrangements, and referral confusion.^9^ The article did not highlight how policy implementers overcame such challenges. Yet, policy implementers occupy a central position in change management practices in the public sector.^19^^,^^20^

Policy implementers have been shown to overcome implementation challenges through local policy arrangements, acting as powerful actors in the public service system. While doing their jobs, public service workers interact directly with citizens and shape the contents and the processes of the policies rather than simply being policy takers.^21^^,^^22^ They are an important part of policy implementation and can cope under pressure, resist change, adopt a paternalistic mentality toward service users, and even engage in policy sabotage.^23^ The decisions they make, routines they establish, and inventions they make to cope with certain pressures related to work effectively translate to or become public policies but do not change the central policy.^22^

Policy implementers have been shown to overcome implementation challenges through local policy arrangements, acting as powerful actors in the public service system.

This policy implementation process is often exacerbated by resource scarcity, such as limited funding or cash flows, lack of staff, and ambiguous and unattainable expectations of performance, which lead to the need to adapt policies locally.^24^ Lipsky perceived that policy is about the service delivered to clients, meaning that public servants, as “street-level bureaucrats,” act as mediators between policymakers and the recipients of services.^22^ Yet, we hardly pay attention to such deeds by policy implementers despite their actions being likely to influence outcomes.^25^ The policy implementer theory challenges “top-down” models of policy creation, which propose that policymakers decide policies delivered by implementers.^26^

The scarcity of resources for the Kenyan FMP has been documented,^9^ but there is a paucity of studies on how the policy implementers bridge the challenges and gaps, especially in implementation. Understanding the local responses characterized by the “distance” between written policy and the reality of implementation in low-income settings is imperative in implementing the FMP. Researchers have highlighted the criteria that policy implementers used to identify poor and vulnerable populations that should be exempted from user fees in Burkina Faso,^27^ how the policy implementers' knowledge has jeopardized the achievement of universal health coverage,^28^ and how health care workers (HCWs) have used informal solutions in Uganda.^29^ To our knowledge, studies have yet to examine the strategies applied by different implementers in the Kenyan FMP; thus, analyzing how they construct public service policies is significant. Therefore, this study sought to explore how HCWs and other local policy implementers used different mechanisms to implement the FMP and made different modifications to the policy.

## METHODS

### Analytical Framework

We address the research questions using Lipsky's^22^ perspective of policy implementers through the empirical analysis of how these implementers act and their role in constructing strategies for public service delivery. Tuurnas et al.^30^ underscored 3 separate but overlapping strategies important in constructing service systems: policymaking, working practices, and professionalism.

The strategies of policymaking involve the policy implementers creating unique developments from policy systems. For example, policy implementers, given the context of their relationship with the users, create their own ethical codes and rules to tackle their workload and tasks efficiently. They act as the go-between the users and the policy developers and translate policy into perceived and concrete actions.

The strategies for shaping the working practice involve the policy implementers taking an active role in defining a new identity for public policy through their daily work. Given the challenges of the policy implementation contexts (e.g., lack of financing and poor monitoring and supervision), policy implementers have the discretion and freedom to innovate the implementation process.^23^

Finally, the strategies of professionalism and ethical tact involve the policy implementers increasing the professional group autonomy as part of the service provision through expert and contextual knowledge to enhance the operations.^23^ Policy implementers often face difficulties in maintaining organizational structures given the challenging implementation context.

**FIGURE 1:**
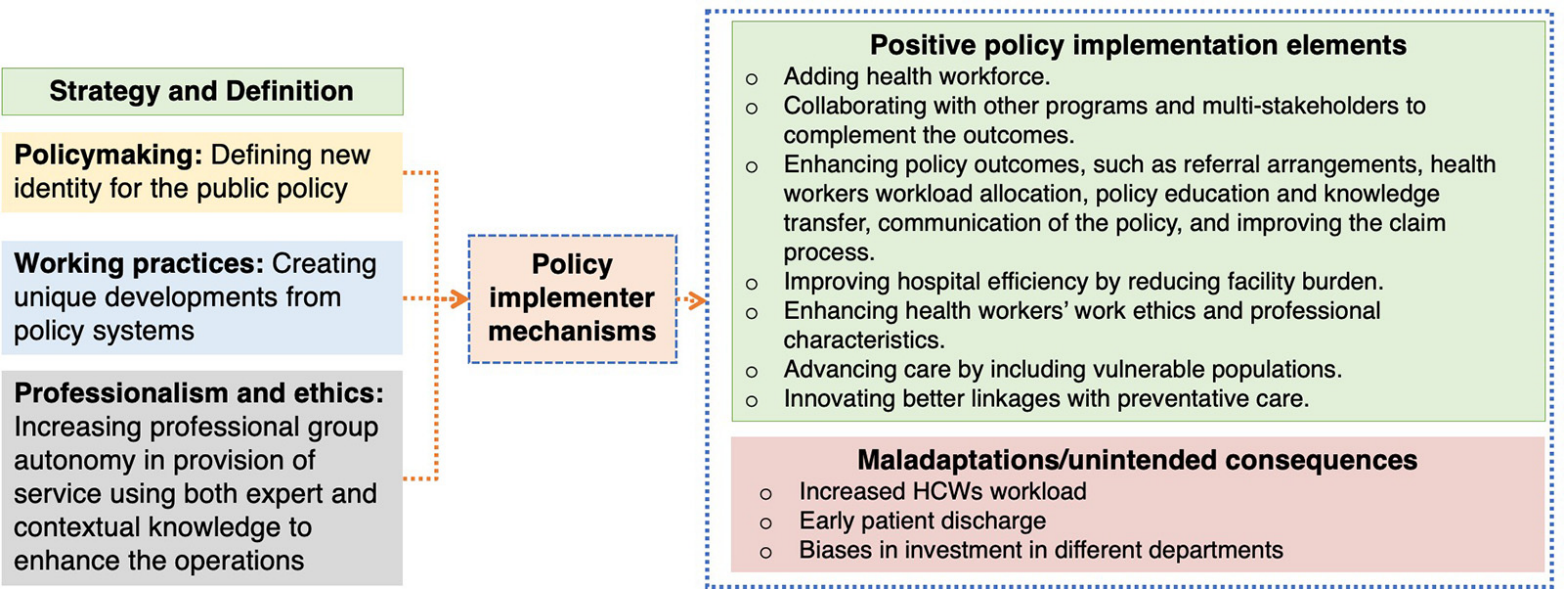
Policy Implementer Strategies Abbreviation: HCW, health care worker.

In the implementation context of the FMP in Kenya, we analyzed the strategies used by the policy implementers who ensured that services were adequately provided (Figure 1). Given the challenges that policy implementers face, their implementation strategies may sometimes produce unintended consequences or maladaptation. These are the reactive subversions, intentionally or unintentionally, implemented by decision-makers at various levels to “hit the target” even though they “miss the point” or to reduce the performance where targets do not apply.^31^ Using this framework, we have also captured some of these maladaptations guided by the data.

### Study Design

The qualitative data reported in this study were extracted from a larger study that the authors conducted to examine the policy process, quality, and cost of free maternal health care in Kenya.^10^ The larger study followed a convergent parallel mixed-methods case study design (involving document review, analysis of nationally representative demographic health survey data, and data from mothers' exit interviews following delivery). This was combined with qualitative exploration using data from key informant interviews with national stakeholders, in-depth interviews with county officials and HCWs, and focus group discussions with mothers who returned for postnatal care. Other aspects of the qualitative data from the larger study have been analyzed, written, and submitted for peer review in other journals. This article focuses on and reports findings from the qualitative data that explored the nature of mechanisms used by the HCWs and the county officials in shaping the implementation of the FMP.^32^

### Study Setting

This study was conducted between November 2018 and June 2019 in 3 purposefully selected public facilities: a high-volume referral hospital (level 5), a medium-volume hospital (level 4), and a low-volume health center (level 3) in Kiambu County, Kenya. The county was chosen due to the logistic feasibility of data collection and the sociodemographic characteristics, health indicators, and population size that have been discussed in detail elsewhere.^33^^–^^35^ The facilities were chosen in consultation with the county team to provide a nuanced understanding of the dynamics of the continuity of free maternity services from the previous policy to the current FMP provided across the different government facilities and to capture unique subcounties' dynamics given their richness in information and characteristics.

### Study Population and Sampling

We purposively selected participants with knowledge of and experience in the implementation of the FMP at the county level (including county and subcounty level officials from the County Department of Health) and the facility level (including facility in-charges, HCWs in charge of offering maternal care/services, and other cadres of hospital workers) (Table). We further purposively selected national-level policymakers as key informants who had knowledge of the FMP as it was designed or envisaged from the onset to ascertain and complement some of the implementer strategies gathered from the first group of respondents. The key informants included participants from MOH, NHIF officials, and development partners (Table).

**TABLE.** Summary of Respondents Interviewed for Insights Into Strategies Used by Policy Implementers for Free Maternity Policy in Kenya

	Female, No.	Male, No.	Total, No.
County and health facility			
County department of health officials (senior-level and middle-level managers)	2	1	3
Public hospital managers (medical superintendent, facility-level managers, department in-charges, and hospital administrator)	7	2	9
Health workers (nursing officers, clinical officers, and accounting/clerical officers)	6	3	9
Total	15	6	21
National			
Ministry of Health officials	4	1	5
National Hospital Insurance Fund officials	3	0	3
Development partners	2	5	7
Total	9	6	15

Before the interviews were conducted, participants were informed about the study's purpose, the right to withdraw, and measures of confidentiality. Participants gave their written and oral informed consent. Participants were informed that data would be reported in an aggregated format and that anonymity would be ensured in storage and publication of the study's findings.

### Data Collection

Data collection was done in 2 stages. The first stage involved in-depth interviews (IDIs) with the implementers (n=21) from the county office and the facilities using semistructured interview guides that were developed to capture the implementation experience of the FMP. The semistructured interview guides' construct validity was tested in a nonparticipating facility to check for ambiguity and flow of the questions. One researcher (BO) conducted all the IDIs at a place of convenience for the participants. The interviews were conducted in English and audio-recorded, and each lasted between 30 and 60 minutes.

The second stage involved IDIs with key informants using a semistructured guide. Two researchers (BO and SO) conducted all the key informant IDIs at a place of convenience for the participants. The interviews were conducted in English and audio-recorded, and each lasted between 45 and 60 minutes.

### Data Management and Analysis

All the IDIs were transcribed verbatim in English and compared against their respective audio files by BO for transcription and translation accuracy. All the validated transcripts were imported into NVivo 12 for ease of management and transparency of the analysis process and organized according to source respondents. The data were analyzed using a thematic approach for its clarity and methodical transparency account of epistemologically neutral coding.^36^^–^^38^ We followed the steps involved in the framework thematic approach^39^ guided by the conceptual framework and the implementer theories described. One researcher (BO) assigned unique identifiers to the data, familiarized himself with the data through immersion, and repeatedly read and reread the transcripts. He then started by developing lower-order premises evident in the text^40^ through open coding (assigning codes to portions of data),^41^ thereby creating an initial coding framework. Study team members (SK and SP) reviewed and discussed the initial coding framework, and any discrepancies were appropriately reconciled. The final coding framework was applied (BO) to the data, and the data were charted to allow the emergence of themes through comparisons and interpretations.

### Ethical Approval

Ethical approval for this study was obtained from the University of Kent, SSPSSR Students Ethics Committee, and AMREF Scientific and Ethics Review Unit in Kenya (Ref: AMREF – ESRC P537/2018). The county and the facilities provided approval to conduct the study.

## RESULTS

The results show that HCWs and county officials applied several strategies that were critical in shaping the policymaking, working practice, and professionalism and ethical aspects of the FMP but also resulted in policy maladaptations.

### Strategies of Policymaking

#### Hospitals Employed Additional Staff

All the facilities in the study reported employing additional staff, specifically clerks, health records information officers (HRIO), and nurses on a locum basis. The clerks and HRIOs supported the registration of mothers and educated them on the importance of the policy. Given that the HRIOs were the first point of contact with mothers at the facilities, they were perceived as being critical in the policy implementation as they were able to shape the mothers' perception, influence them to take up services in the future, and make accurate claims.

As the first point of contact with mothers at the facilities, HRIOs were perceived as being critical in the policy implementation as they were able to shape the mothers' perception and influence them to take up services.

*The most powerful person is the [HRIO] because … if the [HRIO] … gives a negative reference or give a negative perception towards the Linda Mama [revised FMP] then the mothers and maybe society will tend to shy off from getting the insurance.* —Accounting officer

Additionally, the HRIOs and clerks ensured that the mothers submitted the required documentation in a timely manner to avoid missing reimbursement due to strict guidelines by the NHIF. The hospitals provided the clerks and HRIOs with a photocopier to support the mothers who sought services and lacked resources to meet basic costs (e.g., copying IDs and other paperwork as was required).

According to a nursing officer's response, the additional nursing staff employed helped reduce HCWs' workload.

#### County Strategies Alleviated Facility and County Burden

Through the county health management team (CHMT), the county implemented strategies that alleviated the burden on the facilities and the county caused by the FMP. For example, the team gave the health facilities the leeway to use the Linda Mama funds for employing additional workers without requiring further approval from the county offices if this was put in the hospital budget and plans. Additionally, the county lawmakers developed or were developing bylaws to enhance freedom of flow of funds. As a majority of the respondents noted, the flow of funds from the county to the facilities and vice versa was determined by the Public Finance Management Act.^42^ Further, the county lawmakers worked on bylaws that encouraged the ease of access to the funds by the facilities that generated them and the freedom to spend the funds without sending the money to the county revenue fund account.

*Depending on the bylaws [the county] have passed; they have been given autonomy to access the funds directly… they [the county] are able to do a lot with that money, but in some counties, there is no much change.* —NHIF official

However, the act to ensure that facilities spent the money appropriately depended on the governance at the hospital level.

#### Complementary Programs Supported Utility of the FMP Services

Some of the hospitals collaborated with a network of community health volunteers, who were retired nurses in the facilities, to help track pregnant women from the villages when they were due for delivery and ensure they were taken to the health facilities at the appropriate time before delivery. It was unclear whether the hospital paid the volunteers. The CHMT collaborated with the hospitals and worked with community leaders chosen by the community to impart health knowledge and train the community on good maternal and neonatal health care and disease preventive strategies. It was unclear whether leaders received compensation.

In addition, the county, through the CHMT, attracted development partners to support the uptake of Linda Mama services and improved quality outcomes using different approaches targeting elements such as registration, training, enrollment, and communication.

*We've also been able to together with partners we have obstetric scans done free of charge at a facility.* —County senior-level manager

### Strategies of Working Practices

#### Hospitals and HCWs Devised Arrangements for Referring Mothers

Some facilities, especially those that had loyal clients (and had not been able to pay for transport) and who were at high risk for complications at delivery, incentivized the clients by providing them with a means of transport to the facilities during labor.

*[Facilities] were taking matters into their hands, pay for like an ambulance of course which is a taxi.* —Development agency official

These facilities were not being reimbursed for the funds. Additionally, some facilities worked with the CHMT and other partners to reach the mothers in need by providing arrangements that included using each other's means of transport when it was not in use.

*Even when the call center is busy, we have partners like the [General Service Unit], the prison. The [General Service Unit] they always assist us in case we are in dire need.* —Facility-level manager

The facilities agreed upon the reimbursement for such actions.

#### Clerical Officers Created Efficient Procedures

The clerical officers color coded the files to avoid making double claims for both FMP and NHIF services. In addition, they also distributed clerical claims filing work to ensure that only 1 records officer handled the FMP claims for efficiency of work and ensured that all mothers' claims were captured.

#### HCWs and Mothers Were Educated on Policy

Some nurses had those trained on the FMP processes and guidelines conduct interdepartmental fora and workshops, commonly referred to as continuous medical education (CME), to improve HCWs' knowledge of the procedures. These fora were perceived to improve the policy implementation.

The facility in-charges and managers, especially in the referral facility (level 5), invested the hospital finances, particularly those obtained from the FMP reimbursements, toward improving HCWs' knowledge of dealing with complications and referrals.

*We are trying to … make sure that our staff being a referral facility are well trained. We keep on giving them updates so that each person who is working in that maternity knows how to manage these complications that come with, that accompany delivery or that could follow a delivery.*—Facility-level manager

Constrained by the lack of service provision guidelines, HCWs noted that they were going above and beyond by resorting to online learning of the Linda Mama processes.

In addition, the HCWs, nutritionists, and other specialists conducted health talks when most of the mothers attended the clinic to inform them on how to register themselves for the FMP, family planning, and expectations during delivery.

#### The County and Hospitals Adopted Innovative Communication Procedures

**FIGURE 2:**
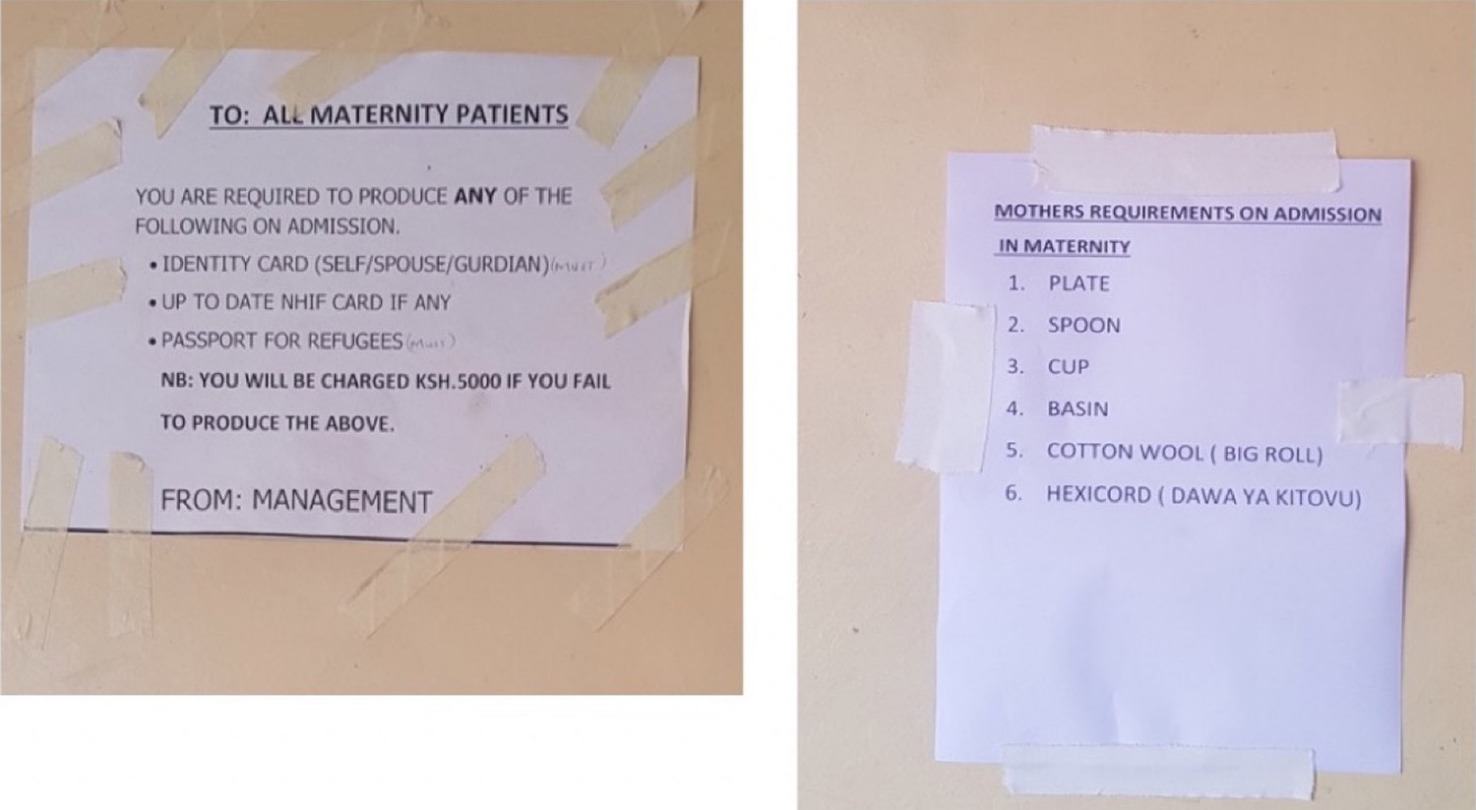
Example of Posters Used in Some Kenyan Public Health Facilities to Inform Clients of Free Maternity Policy Registration Requirements

In all the facilities, staff created posters that educated the mothers on registration requirements for the Linda Mama policy. To ensure that the mothers provided all the required documents (such as IDs or passports) for registration and avoid claims rejection by NHIF, some facilities communicated that mothers would be fined if they did not meet some admission requirements, even though the policy was free (Figure 2). Some of the strategies were promoted by the senior managers at the county to the HCWs.

Additionally, the NHIF used communication providers (e.g., Safaricom and Airtel) to ease the process of registration by using phone networks rather than paper.

#### Hospital Clerical Officers Collaborated to Ease the Claims Process

The hospital clerical officers who worked on the FMP claims collaborated closely with both the NHIF county office and the hospital maternity department to work on the Linda Mama claims and ease other processes of work.

*NHIF … always work on our claims…Sometimes they call us for a meeting … to raise our complaints. They will also … give us their views so that we can work as a team. So, there is always a way out to work for our claims. And we also have their numbers in case of anything we complain.* —Clerical officer

If the NHIF office had reverted to the clerical officers with corrections on the claims, the officers worked with the maternity wing nurses and employees to get the correct information from the mothers' files, which facilitated follow-up and correction of claims.

*Sometimes I'll take some claims and maybe they will say that they have some mistakes. … So, they return the claim … I just call the maternity and find out the document on where the mistake was*. —Clerical officer

#### Smaller Facilities Collaborated With Bigger Facilities in Claims Processes

Because of the logistical and resource challenges related to claims (e.g., fewer staff to handle claims, lack of knowledge, and lack of computers), smaller facilities collaborated with bigger facilities to ease the claim process. For example, health centers that offered FMP services sent their data through bigger hospitals to ensure that their claims were made as if they were a higher-level facility.

*[Small facilities] are offering [FMP], but they are not claiming [because of] few staff [or] they don't have somebody for logistics and claiming. Some of them like in [the county] were innovative … when they deliver, they send their data through a hospital. They claim as if they were maybe a level 4, all the data is claimed through the major hospitals because that is where maybe the system has been there. So, I think the funds flow now to these facilities.* —MOH official

The county, through the CHMT, also supported the smaller facilities to make claims on their behalf to not lose revenue, and, in turn, because the FMP was considered a form of county revenue, the county did not lose funds.

*NHIF focal person in [the county] is to make sure that they [smaller facilities] get their service as quickly and as efficiently as possible. The other issue is to make sure that we've claimed on behalf of the facilities because it's a form of revenue for [the county].* —County middle-level manager

### Strategies of Professionalism and Ethics

#### Nurses Provided Additional Support and Advocacy to Mothers

Some nurses reported that they registered and advised pregnant mothers as part of their daily activities apart from their work. They used it as an opportunity to teach the mothers about Linda Mama and eventually helped them choose the appropriate facilities for delivery.

Some nurses reported that they registered and advised pregnant mothers as part of their daily activities apart from their work.

*Like yesterday I was [at] a salon and I saw a pregnant woman and I asked her, “Have you heard of Linda Mama?” She told me “No,” and I registered her. Everywhere I go … I go registering mothers. . . . The other day I registered some at Eastleigh [a subcounty in Nairobi county] and some of them have never heard of Linda Mama.* —County senior-level manager

Nurses and other HCWs worked over and above their duties to ensure pregnant women were referred safely.

*We … take that it is our work to refer them especially when the mother comes in active stage we can't refer to go alone we have to escort them.* —Nursing officer

Equally, the nurses disclosed that they also followed up on the outcomes of the mothers' after referral.

*In [referral facility C], the patients we have referred … they always give us a feedback. Especially when it comes to maternity, antenatal, and newborn.* —Facility-level manager

Although it was part of the nurses' job to refer mothers, they kept proper records on maternal cases, including those they referred, and ensured the referred mothers got the required care in addition to escorting them at the time of referral.

#### Hospitals Included the Nurses in Planning and Budgeting

Some hospital budgeting and management committees involved the nurses in planning the funds. By giving nurses some sense of authority and freedom in the hospital budgeting process, the hospitals empowered nurses to decide how to spend the funds, as they did the work that brought the funds. The committees and hospitals perceived it as being professional. Although the County Chief Officers of Health could suggest areas of spending, the authority to incur expenses came from the county level. The facilities' committees planned, budgeted, and decided to involve the nurses in the processes as well.

#### HCWs Provided the FMP Services to Those Excluded From the Policy

In the Linda Mama manual, the inclusion and exclusion criteria for beneficiaries excluded foreigners. However, in all the facilities, the HCWs provided care to all mothers who came to the facilities.

*So irrespective of whether they have the requirement, the required document or not, services will be offered so we talk later. And you will find cases where if worse comes to worst you have to still release the mother without those documentation, they cannot be able to pay.* —Facility-level manager

Because of their location, some facilities received a high number of foreigners who did not have identification and provided them with free maternal services without asking for payment.

*[Facility A] we have many foreigners from Uganda. So, some come, some they don't even know the importance of clinic. So, they don't attend the [antenatal care] clinic they come the last minute.* —Nursing officer

Despite the requirement of needing an ID before delivering the service, the facilities sought ways of bridging the gap for those without IDs, including children who had no parents, refugees without IDs, or schoolgirls who were underage and pregnant. For example, to make a claim for the services provided to some vulnerable groups, the HCWs used any person's ID (who was not necessarily pregnant) to ensure that no mother paid for any service and that the facility did not lose Linda Mama claims. For underage clients, the HCWs resorted to using the guardians' IDs. If the guardians were not available, HCWs provided the services for free and bore the cost.

*If a person doesn't have an ID, we ask them to use even their mother's ID or their auntie's ID, just any other lady in their lives' ID. Because … we want to make sure that every mother is served, every mother is not forced to pay anything until they get out of the maternity with their babies.* —Clerical officer

*There is that allowance for … the underage kids … who deliver. . . . but they are maybe probably in school … we require a letter from the school to show for sure that they are a student and also the guardian or the parent brings the ID. . . . probably there are some who are married underage, but you cannot use the spouse ID because ideally an underage cannot be married. So, we use the guardians' or the parents' ID to process the same.* —Facility-level manager

For adolescents, people with no literacy, and people with disabilities, HCWs provided education and adolescent services and waived the costs.

*Mostly the challenge is education because the people without education they shy away. You ask for the documents, but they shy away from it … So, it takes a lot of educating for that group. … The ones that come and we realize there is a challenge, we sit down with them and we explain exactly why Linda Mama is important.* —Facility-level manager

By providing services to vulnerable populations, the hospitals bore the costs and used other means to offset them, such as through the government social work waiver system.

By providing services to vulnerable populations not included in the policy, the hospitals bore the costs and used other means to offset them.

*We are a government hospital, we don't turn anybody away. Even if they come and they don't have Linda Mama you still take care of them. But now it is on a waiver system, you waive the cost … Because there is nowhere you will claim the waiver … Because the funding is either Linda Mama and NHIF or [Facility Improvement Fund (Cost sharing)]. So, if they can't pay through all those channels, it's waivered and the hospital absorbs the costs.* —Facility-level manager

In some facilities, because of the rigidity of the claim service, they provided the service and let the mothers go, as the mothers were not concerned about the claim system but only about receiving care.

*The patients themselves are not too much interested in which fund you use to fund their care. All they want is care and go home. Now to claim under the insurance cover requires a lot of personal involvement, IDs, filling of forms, declarations, and authorizations. So the hospital is left right in the middle, you cannot ask for the cover. But have to use the underfunded one.* —Facility-level manager

#### Facilities Invested in Preventative Health Care

Some facilities added noncommunicable diseases screening centers (e.g., blood pressure and blood glucose monitoring) to ensure fewer mothers were admitted to the medical and surgical wards with life-threatening noncommunicable diseases and complications would be identified early. Eventually, with fewer noncommunicable disease cases admitted, the HCWs in those wards were relocated to reproductive clinics to provide services to mothers.

*…To help in making better our maternal health services, we've been able to major on the preventive like having noncommunicable disease centers. So that we can have, less patients admitted in the medical and surgical wards, so that the staff who are there can be deployed in reproductive health settings to be able to give quality services.* —County senior-level manager

### Maladaptation of the Policy

#### HCWs' Workload Increased

Facilities (especially those with no additional locum staff) made HRIOs work extra hours to capture as many mothers as possible, which translated to claims and reimbursements to the facility. Even though this practice was intended to enhance policy implementation, it likely had negative, unintended effects, such as staff burnout, which was counterproductive.

Some facility managers reported that some nurses sought transfers to lower-level facilities to avoid the heavy workload in higher-level facilities caused by the FMP.

*Most of the nurses do not want to be deployed to go to the health center because the workload is too high. . . . Most of the workers want to go to the level 2s that's the dispensaries and other health centers where the workload is not as much.* —Facility-level manager

The facility in-charges noted that the hospitals incurred debt for meeting the costs of the additional staff because of delayed reimbursements.

*Most of the times we have locum arrears, like even for 3, 4 months … and even when that money comes sometimes you can't pay, you can't clear the arrears because there are things also to cater for.* —Facility-level manager

#### Partners Did Not Equip Facilities Equally

One partner equipped some lower-level facilities with an ultrasound.

*We have sonographers … we have empowered the level 3s to be able to conduct deliveries. You know long before people would say that if it is your first delivery you don't deliver in a health center … [a facility with partners incentive] does around 250 scans per month and it's free of charge. You know that is a level 3.* —County senior-level manager

Although the availability of the ultrasound in the lower-level facility was perceived to be a benefit, it increased the number of users at the facility and skewed the distribution of HCWs (more HCWs in the facility with the ultrasound than in those without). It also resulted in referral from higher-level facilities that did not have an ultrasound to the lower-level facilities with the ultrasound.

#### Facility Managers Prioritized Maternity Services

Although supplies purchased through reimbursements from the FMP were equitably distributed across all departments in all facilities, it was noted that the in-charges and the HCWs prioritized both hospital workload balance and investments in maternal care from the Linda Mama policy reimbursements.

*We give priority to maternity … when we get any extra staff, we send them to maternity so that we try to boost their numbers.* —Facility-level manager

More investments were directed to the maternity wing by restricting other departments of funds because it brought more funds through the FMP reimbursements. In addition, mothers under the FMP were given priority compared to other hospital patients so that the wing did not incur any costs.

*We are forced to give [patients] priority because we don't have an option of sending the patient or letting the patient bear the cost. So, it would adjust the budgeting lanes, it will give them [maternity wing] priority in limited resources. It would also adjust the supply lines to skew it a little more towards giving the mothers free maternity care … we actually plough in back all of it and squeeze other departments to add up.* — Facility-level manager

#### Mothers Were Discharged Early

Some facilities discharged mothers immediately after birth, especially those who had delivered vaginally and whose delivery costs are not perceived as high, even before offering the fully costed FMP benefits, to avoid incurring additional costs. Before this strategy was implemented, facilities had to keep the mothers to receive the payments, but later, the facilities did not keep mothers in facilities because it did not determine being paid for by the policy. Eventually, with a full economy of scale, the facilities maximized their profits, but it reflected that the reimbursements needed to be adequate to meet the full costs of skilled delivery. The unintended consequence of this early release was that the few mothers who had complications and may not have received adequate treatment experienced further complications.

*If I go to [a referral hospital] today to deliver and I am a mother and maybe 80% of us who go there deliver normally, if I deliver normally, they will take care of the mother that day, the following day, by evening they discharge. So, first of all this mother may not have taken that high cost and two they are not holding mothers anymore, like they were holding before waiting for them to pay. There are no waivers and the exemption that you need to do, there are not holding patients in the ward anymore and adding to the cost, you just release. So even 1 or 2 that complicates, unless the facility does not want to use the same money to serve this client, there was not really that need to make it a big conflict.* —MOH official

## DISCUSSION

This study provides imperative insights into the implementation (and translation) of the FMP into practice and the ability of policy implementers to deliver a government policy. We see a convergence of efforts of both the HCWs and the county teams (as implementers) to define the FMP by implementing strategies, not outrightly written on paper, to ease the burden of facilities and counties caused by the policy. This convergence portrays the roles of county officials working in tandem with HCWs acting as implementers to transform the FMP to fortify service delivery by enhancing its flexibility.

The policy implementers, as “policymakers,” are the prime movers in constructing and fortifying the FMP because they create unique development (rules and content not on policy paper) for the services under the policy. For example, in the contemporary context of the Kenyan FMP, such collaborations and coalitions are useful in developing complementary programs (such as using community health volunteers and attracting strategic development partners), that have also been observed in other Kenyan free policy evaluations.^43^ Formulating bylaws to enhance the freedom of flow of the funds allows hospitals to use the funds directly. Literature on other funding schemes directed to facilities has shown that, if given proper oversight, these schemes can strengthen performance and improve HCWs' motivation,^44^ and as such, these bylaws can strengthen performance. Further, by employing additional staff, policy implementers adapted their work to their desire, influenced service outcomes such as increasing claims, determined the perception of use, and eased the workload, thereby supporting the utility of the FMP services and enhancing motivation. These staff offer continuity of care and shape clients' service experiences through a “not-so straightforward” system.^45^

Our findings suggest that policy implementers, by implementing content not on policy paper, defined a new identity for the FMP. The HCWs, as implementers, interpreted and adapted the policy in their understanding and in meeting their outcome obligations, as was the case for the South African user fee policies.^46^ Lipsky^22^ showed that service delivery implementers have substantial jurisdiction over how they manage their work but that legislators and policymakers rarely recognize this power. For example, by insinuating that mothers would not be able to get services if they did not provide IDs, the facilities used what Steven Lukes recognized as “power as thought control”^47^ that influenced preference and shaped clients' thoughts. Overall, the communication partly ensured that NHIF rejected fewer claims because it asserted compliance.

By introducing working practices, such as developing different arrangements for client referrals, the implementers corrected the confusion caused by the lack of clarity on referrals, as Orangi et al. reported.^9^ Educating mothers on the FMP aimed to better equip mothers to prioritize their needs and take responsibility for their care. HCWs' participation in self-directed training on maternal procedures aimed to equip them to advocate for clients. Perhaps this training influenced policy implementation, as previous research in Burkina Faso showed that lack of HCW knowledge contributed to inequity in free policy implementation.^28^ By reconstructing the nature of their work in the desired ways, implementers altered the practice model to ensure continuity of service provision and ease the claims process, such as when smaller facilities collaborated with bigger facilities or the maternity department collaborated with the county NHIF teams. Such informal partnerships and collaboration have been shown before as being necessary to provide services.^48^ Clerical officers adopting color coding of files helped identify mothers and expedite the claims process.

By introducing working practices, such as developing different arrangements for client referrals, the implementers corrected the confusion caused by the lack of clarity on referrals.

Bound by ethics and professionalism, HCWs performed beyond their duties to ensure that vulnerable populations (e.g., foreigners, adolescents, and poor) received services. This included working in tandem with the mothers as policy users to holistically cocreate better policy models. However, by advocating for vulnerable populations, HCWs not only increased their obligation and ability to exercise discretion using expert and contextual knowledge to enhance the operations^23^ but also potentially burdened the facilities with additional claims. This finding reinforces the concept that HCWs' working environment influences them as implementers and the sociopolitical context, as well as their personal values and beliefs.^49^ For example, the untimely or inadequate reimbursements and inadequate supplies pushed HCWs to prioritize the few supplies for the most deserving mothers. Choosing which mothers adequately required the services may have been challenging or subjective. Mothers may have altered their dress to tatters to be identified as poor, which may not have been true. Such implementer schemes have led to unintended consequences.^45^ For example, in Senegal, HCWs used their power in the user fee policy scheme to institutionalize discrimination rather than help the clients.^50^ However, for the exempt user fee policy in Burkina Faso, midwives developed criteria (not part of the policy) to help identify indigents and provide them with service.^27^ Therefore, the concept of professionalism shows that HCWs, as policy implementers, make daily situational choices that require improvisation as guided by tacit knowledge and values rather than policy rules.^51^ By being flexible with the policy, the implementers were making it equitable and effective,^23^ hence bridging the gap between policymaking and practice.^52^

Despite offering potential benefits for implementers and their beneficiaries, these adapted practice models may have unintended negative consequences for the implementers, such as additional HCW workload, potentially leading to burnout, which drives HCWs to scheme the system as they seek only to work in centers with a lighter workload.

Additionally, facilities are incurring debt to hire additional HCWs. The mismatch between service demand and available resources to provide services has been documented elsewhere^53^ but should be aligned through timely disbursement of funds if the country is to meet the universal health coverage goals. Also, by prioritizing the utility of reimbursements from the Linda Mama policy to improve maternity areas and develop strategic collaboration between different hospitals and departments, implementers may focus on attracting more clients. This effort results in more claims and reimbursements for the hospitals but also increased workload and diversion of funds from other departments. This finding aligns with a scoping review on free schemes that showed that in South Africa and Ghana, HCWs as implementers respected free policies as long as they had the needed resources to support them.^54^

Although some strategic partners have good intentions in providing facilities with equipment, this causes facilities to be unequally equipped and HCWs to be unequally distributed. Having a strong, coordinated, and well-elaborated agreement between partners and county leadership is imperative.

Our study suggests that scaling up the capacity and resources of policy implementers to manage a public health emergency may be highly strategic, especially in countries with a limited central government response. Additional research is needed on HCWs and county officials as policy implementers, flexibility and agility of the FMP and organizations, and resilience of organizations and the implementers' capacities. The analysis of professional resilience in public services planning needs further theoretical and empirical attention. HCWs' and clients' roles in accountability aspects (horizontal and vertical) need to be more fully cultivated.

## CONCLUSION

This study has elucidated the experiences of the policy implementers overcoming policy challenges by adopting strategies that address those challenges. Some strategies involved implementers reformulating policy, changing their ways of working to achieve policy objectives, and enhancing professionalism and ethics to deliver better services. Workplace strategies created positive policy impacts, such as increasing health workforce employment and encouraging collaboration between programs and different policy players to improve maternal outcomes. By applying professionalism and ethics, the implementers advanced care by including vulnerable (often forgotten) populations and innovated better linkages with preventative care. However, the strategies have also resulted in maladaptation and unintended consequences, such as more workload, early patient discharge, and investment biases.

## Notes

**Peer Reviewed**

**First published online:** October 18, 2023.
